# Do specialist haemoparasites induce tolerance in their hosts?

**DOI:** 10.1017/S0031182025000393

**Published:** 2025-04

**Authors:** Cameron Armour, Abigail L. Hone, Jenny C. Dunn

**Affiliations:** 1 School of Life and Environmental Sciences, Joseph Banks Laboratories, University of Lincoln, Lincoln, Lincolnshire, UK; 2 School of Biology, University of Leeds, Leeds, West Yorkshire, UK; 3 School of Life Sciences, Keele University, Newcastle-under-Lyme, Staffordshire, UK

**Keywords:** generalist, *Haemoproteus*, Passerine bird, spillover, transmission, virulence

## Abstract

Generalist and specialist parasites are predicted to trade off transmission efficiency with host virulence, depending on host range. However, very few empirical studies test this trade-off in parasites at both ends of this spectrum simultaneously. Here, we examine parasitaemia (as a proxy for transmission efficiency) and virulence (assessed through 2 metrics of host immunity) in birds infected by a generalist lineage of *Haemoproteus*, comparing these with birds infected by more specialist *Haemoproteus* lineages, and birds uninfected by any haemoparasite. We compared the same metrics for specialist-infected birds, depending on whether a species was a ‘usual’ host or ‘spillover’ host. Immune metrics of specialist-infected birds did not differ from those of uninfected birds, but generalist-infected birds had elevated heterophil:lymphocyte (H:L) ratios and elevated white blood cell (WBC) counts compared to both other groups. Parasitaemia of specialist lineages was nearly 5 times higher than that of generalist lineages. Moreover, specialist-infected spillover hosts had higher H:L ratios and higher WBC counts compared to usual hosts for these lineages, with parasitaemia nearly 10 times lower in spillover hosts compared to usual hosts, although sample sizes of spillover hosts are, by definition, small. Our data provide support for the evolution of tolerance in specialist host-parasite interactions, with increased transmission efficiency for the parasite and reduced impacts on the host.

## Graphical abstract

**Figure:**
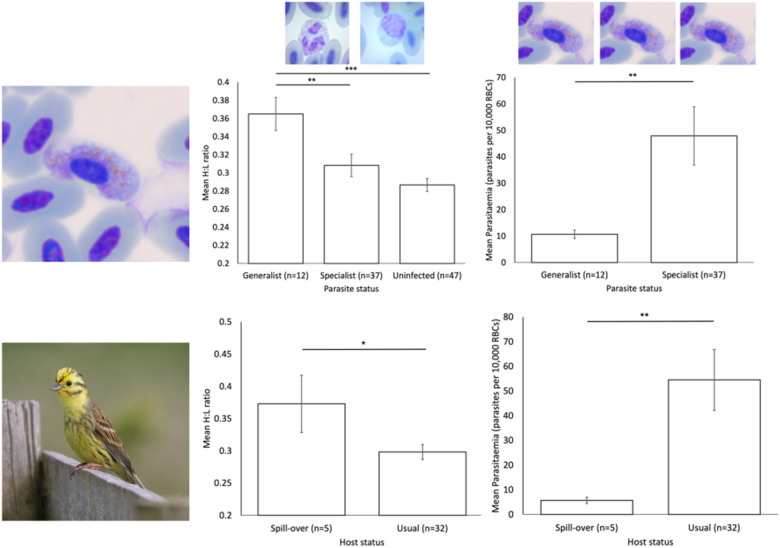

## Introduction

Parasites fall on a spectrum between generalist and specialist, depending on the range of host species from which they can be successfully transmitted (Leggett et al., 2013). The position of any parasite on this spectrum is influenced by a fundamental trade-off between parasite fitness and virulence, but quantitative comparisons demonstrating this trade-off in relation to host range are rare. Fitness is effectively the reproduction rate of a parasite and refers to how successfully a parasite can increase its transmission rate to new hosts (Leggett et al., 2013). Virulence is the harm to the host directly due to parasitic infection: a more virulent parasite reduces the transmission period (or infectious period) by increasing host mortality (Leggett et al., 2013). Together, they affect the fundamental life-history trade-off of parasites in different ways: fitness is an important factor affecting transmission rate, whereas virulence can determine the length of the transmission period (Lipsitch and Moxon, 1997), and the optimal level of virulence is the one that maximizes total transmission (Leggett et al., 2013). Host range and the relationship between parasite fitness and virulence are intrinsically linked.

A wide host range can increase the frequency of successful transmission events, but these generalist parasites are predicted to have comparatively lower fitness and reproductive success within each host species (Garamszegi, 2006): unlike specialists, they may not be able to adapt optimally to the internal environment of their host species (Gandon, 2004). Contrary to this prediction, evidence from avian haemoparasite systems suggests that generalist parasites are those that are able to reach high prevalence within compatible host species (Hellgren et al., 2009), although data on parasitaemia – which is a proxy for reproductive success in parasites such as malaria – in this context are few. For vector-transmitted parasites such as avian malaria, this high prevalence of generalist parasites may be driven by a high encounter rate with infected vectors when a generalist lineage is at high prevalence within a bird community, negating the potential low transmissibility of infection within each host (Hellgren et al., 2009). However, it is important to note that the definition of a parasite as specialist or generalist may also be context-dependent.

Specialization to one or a small number of closely related hosts species results in a reduced ability to infect and be transmitted from other host species (Hellgren et al., 2009), which can be compensated for in 2 ways (Frank, 1996). Specialist parasites could select for higher replication rate, increasing the frequency of transmission events but incurring greater fitness costs on the host, resulting in a shortened transmission period, known as the trade-off hypothesis (Frank, 1996; Lievens et al., 2018). Alternatively, they could select for lower replication rate to prevent overexploitation of the host and extend the transmission period at the cost of a reduced transmission rate, known as the niche breadth hypothesis (Leggett et al., 2013). Two studies in particular have specifically addressed this question, with contrasting results: a study focussing on house sparrows *Passer domesticus* and supplementing these data with global data on parasite-host relationships to define parasite host breadth, found support for the niche breadth hypothesis (Cebrián-Camisón et al., 2024). In contrast, a study of 4 geographically distinct host–parasite communities fond no strong support for either hypothesis (Drovetski et al., 2014). In other systems, such as primate malarias, research suggests that specialist parasites have greater virulence as quantified through peak parasitaemia, which is linked to increased host mortality in this system (Garamszegi, 2006). However, within avian malaria, there is some evidence that hosts may be able to evolve tolerance to these parasites, consequently reducing virulence (e.g. Atkinson et al., 2013), although this is an area where more research is required (Rivero and Gandon, 2018). Thus, impacts of specialist parasites on their hosts may depend on whether they infect their usual host species or spill over into an alternative host (Galen et al., 2022).

Specialist and generalist strategies can be influenced by a range of host and environmental variables and may depend on the contexts of host abundance and geographic range. For example, generalism tends to be associated with wider geographic ranges, possibly due to the wider availability of potential resources (host species; de Angeli Dutra et al., 2021). Host abundance may also influence parasite specificity such that hosts of specialist lineages tend to be more abundant (Svensson-Coelho et al., 2016), although consistent support for this is weak. Parasite specificity is associated with higher prevalence in some systems (Garcia-Longoria et al., 2019) but not others (Hellgren et al., 2009). A general pattern of multihost parasites using closely related host species suggests that many associations result from co-evolutionary relationships (Clark and Clegg, 2017; Ellis et al., 2020), leading to the potential for immune-modulated drivers of prevalence within host species (Garcia-Longoria et al., 2019). Intriguingly, different strategies may be employed by the same parasite in different scenarios and in different geographic locations (Ellis et al., 2020; Valkiūnas et al., 2024); thus, it is important to take into account the local strategy employed by a parasite with the study system of interest.

Specialist and generalist parasites are both capable of infecting hosts outside their typical host range. In the case of specialists, this can be referred to as a ‘spillover’ host, with their target species being their ‘usual’ host (Leggett et al., 2013). Spillover hosts of specialist parasites, especially, tend to be closely related to the parasite’s usual hosts (Schatz and Park, 2023). Since a specialist will not have coevolved with a spillover host, it could suffer fitness costs due to a lack of adaptation to survive and replicate in an unfamiliar host species (Ebert, 1998), although some evidence suggests that transmission potential may be higher in spillover hosts (Auld et al., 2017). Additionally, there may be a difference in the magnitude of impact on the host that is triggered as a result of infection within a spillover host compared to a usual one (e.g. Farrell and Davies, 2019; Ortega et al., 2022). For example, parasites infecting hosts outside their usual phylogenetic host range are more likely to result in lethal infections (Farrell and Davies, 2019), which may be driven by phylogenetic distance from the usual host (Galen et al., 2022).

Avian host–haemoparasite systems are excellent models for studying ecological and evolutionary questions in the animal kingdom (Rivero and Gandon, 2018; Dunn and Outlaw, 2019). *Haemoproteus* is a vector-borne haemosporidian parasite that is widespread and abundant in wild passerine populations and transmitted by louse flies (Hippoboscidae) and biting midges (Ceratopogonidae; Valkiūnas, 2005). *Haemoproteus* can potentially be highly virulent and lethal for avian hosts (Ortiz-Catedral et al., 2019); however, the majority of infected birds are asymptomatic, and infection has, in some cases, been linked to increased host fitness (Zylberberg et al., 2015). Here, we take advantage of a community-level dataset of passerine–haemoparasite interactions to identify generalist and specialist parasite lineages (Woodrow et al., 2023). We examine blood smears from (1) birds infected by a generalist parasite lineage; (2) both usual and spillover birds infected by specialist lineages and (3) uninfected controls from the same host species; to quantify 2 metrics of immune function (as a proxy for virulence), and parasitaemia (as a proxy for transmission efficiency). *Haemoproteus* is an ideal parasite to use as a study system here because only the transmissible gametocyte stages are found in the circulating blood, suggesting that parasitaemia should be a good proxy for transmission efficiency. We use these data to test 2 specific hypotheses:

1. Birds infected by a generalist *Haemoproteus* lineage have a larger immune response than those infected by a specialist lineage, compared to uninfected controls, coupled with higher parasitaemia.
2. Spillover hosts for specialist *Haemoproteus* lineages have a larger immune response than usual hosts, compared to uninfected controls, coupled with higher parasitaemia.

## Materials and methods

### Sample collection

Blood samples were collected from a range of passerine birds as part of a wider study (Dunn et al., unpublished data; Woodrow et al., 2023) during April–August, 2017–2019. Birds were caught using mist nets at 4 sites within 10 km of the city of Lincoln, UK (53°13ʹ48”N, 0°32ʹ27.0”W; Woodrow et al., 2023). Sites consisted of patches of woodland and scrub, within an arable farmland landscape. Once caught, birds were aged and sexed where possible by reference to plumage characteristics (Svensson, 1992) and had a small blood sample taken from the brachial vein using a sterile needle and a capillary tube. Two blood smears were created for each bird, and the remaining blood ejected into an Eppendorf tube before being frozen at −20°C within 8 h of collection. Blood smears were fixed using methanol within 8 h of collection, stored at 4°C for up to 1 month until they were stained using Giemsa stain. All samples analysed here were collected between April and July 2018; we used data from 2017 to 2019 to define our host and parasite lineages (next section).

### Host and parasite lineage definitions

We base our definitions here on observations from only our local study site including data from 2017 to 2019, rather than on global patterns of host–parasite associations because elsewhere we observe that host–parasite associations may differ spatially (Woodrow et al., 2023). Based on previous polymerase chain reaction (PCR) analysis of the same samples (e.g. Woodrow et al., 2023), we selected samples from individuals with known infection status based on their infecting parasite lineages, rather than host species, including 8 parasite lineages. We only chose birds with single infections for this study and ensured that samples for each lineage were spread across ages and sexes of birds where possible because age and sex can influence haematological parameters (Hernández and Margalida, 2010).

Generalist and specialist parasites are generally defined using host specificity indices (e.g. Poulin and Mouillot, 2005; Hellgren et al., 2009), which take into account both the relatedness of different host species and the prevalence of a parasite lineage within each host species. Here, we only sample passerines, and the majority of sampled hosts are from different genera, so accepted indices of host specificity, which take into account host relatedness and parasite prevalence (within each host species, rather than the proportion of infections represented by each host), do not clearly reflect patterns we observe in our data. Consequently, as we are interested rather in the host diversity in terms of the evenness or dominance of host species for each lineage, we use a Shannon diversity index (Shannon and Weaver, 1949) to distinguish between generalist lineages (where many host species are used but none appear favoured) and specialist lineages (where one host species is the most commonly used host, but other hosts may be infected rarely). We here define a generalist lineage as one with a Shannon index >1 (high number of hosts and high host evenness) and a specialist lineage as one with a Shannon index <1 (low number of hosts and/or low host evenness; see Supplementary Table 1). For completeness, we also include measures of phylogenetic distinctiveness (SPD_i_) and phylogenetic diversity (PD_i_) in Supplementary Table 1, both of which take into account the phylogenetic relatedness between multiple host species (Poulin et al., 2011). SPD_i_, which is independent of the number of host species used by a parasite and uses distance matrices to control for phylogenetic distinctiveness, was calculated using the *taxondive* function in the *vegan* package (Oksanen et al., 2017) in R (R Core Team, 2024). PD_i_, or Faith’s phylogenetic diversity, incorporates host species richness into the genetic diversity index and was implemented using the *pd* function in the *picante* package (Kembel et al., 2010), with a phylogenetic subset downloaded from birdtree (Jetz et al., 2012). Whilst all 3 metrics are highly correlated, we retain the Shannon index for defining our parasite lineages.

For specialist parasites with low host evenness, we wanted to further distinguish hosts within which a parasite was commonly found (‘usual’ hosts) from hosts within which a parasite was rarely found (‘spillover’ hosts). Here, we define a usual host species for any parasite lineage as the host species within which >75% of infections by that lineage occurred; any host species accounting for <10% of infections by a lineage is considered to be a spillover host.

### Microscopy methods

Data were collected from 188 blood smears collected from 97 passerine birds. Host distributions for each lineage are shown in Supplementary Table 1: in total, 12 were infected by the generalist lineage CARCHL01, 37 were infected by specialist lineages and 47 were uninfected, as previously confirmed by PCR analysis and subsequent sequencing (Dunn *et al.*, unpubl. data). From those infected by specialist lineages, 5 individuals were considered spillover hosts for their infecting lineage, and 32 were considered usual hosts.

Each blood smear was examined under oil immersion at ×100 magnification until at least 10 000 red blood cells (RBCs) had been examined for the presence of parasites, and 100 white blood cells (WBCs) had been identified. We subsequently calculated heterophil:lymphocyte (H:L) ratio as number of heterophils/(number of heterophils + number of lymphocytes); WBC:RBC ratio as estimated number of RBCs examined/number of WBCs examined × 100; and parasitaemia as the number of parasites found divided by the number of RBCs examined, multiplied by 10 000.

### Statistical analysis

Statistical analyses were carried out using linear mixed effects models with Gaussian error structure, constructed using the *lmer* function in the lme4 package (Bates et al., 2015) in R version 4.0.5 ‘Shake and Throw’ for Mac (R Core Team, 2024).

All response variables were square root transformed to meet model assumptions of the normality of residuals. Each of the 3 response variables was tested in 2 models to test for differences between parasite status (generalist, specialist and uninfected) and host status (usual or spillover), respectively. Significance of fixed factors was determined using an analysis of variance comparing the model with and without the fixed factor of interest, and parameter-specific *P* values to determine where significant differences lay between groups were calculated using the Satterthwaite method, in the *lmerTest* package (Kuznetsova et al., 2017).

**Figure 1.**
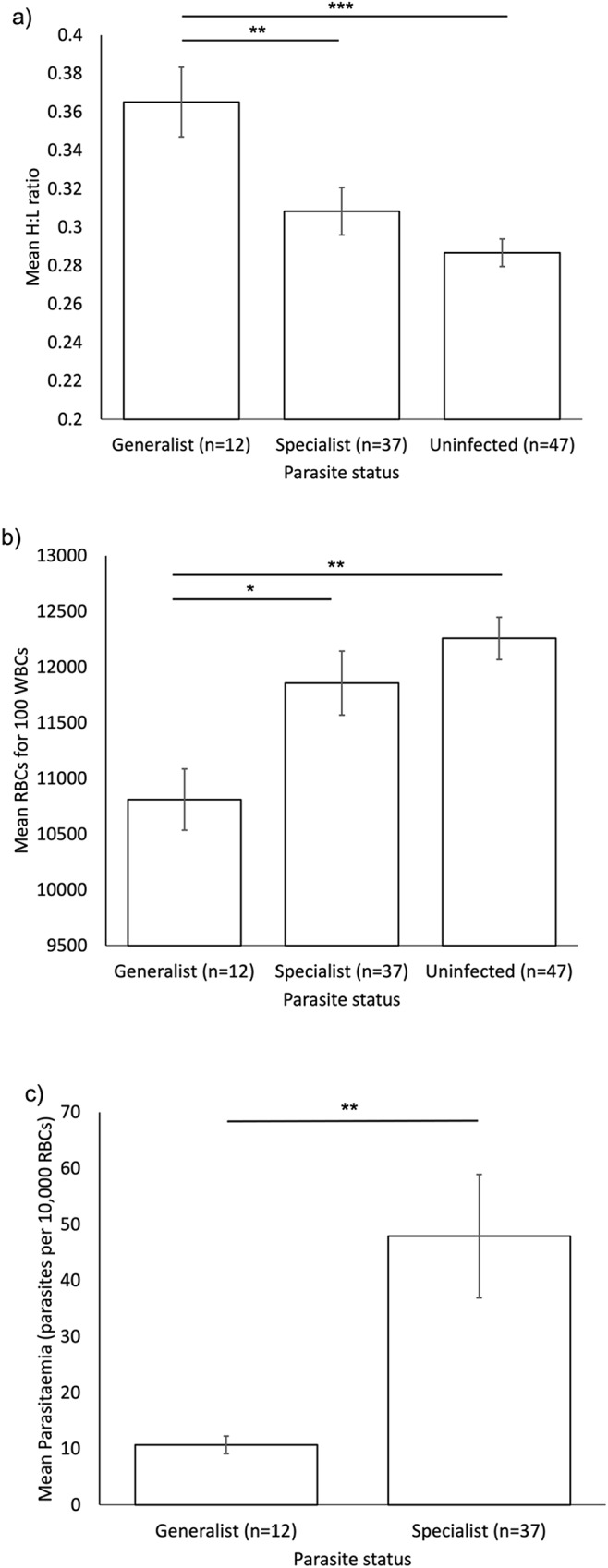
Differences in (a) H:L ratio, (b) RBC:WBC ratio and (c) parasitaemia for birds infected by generalist and specialist parasite lineages, and for (a and b), uninfected birds.

**Figure 2.**
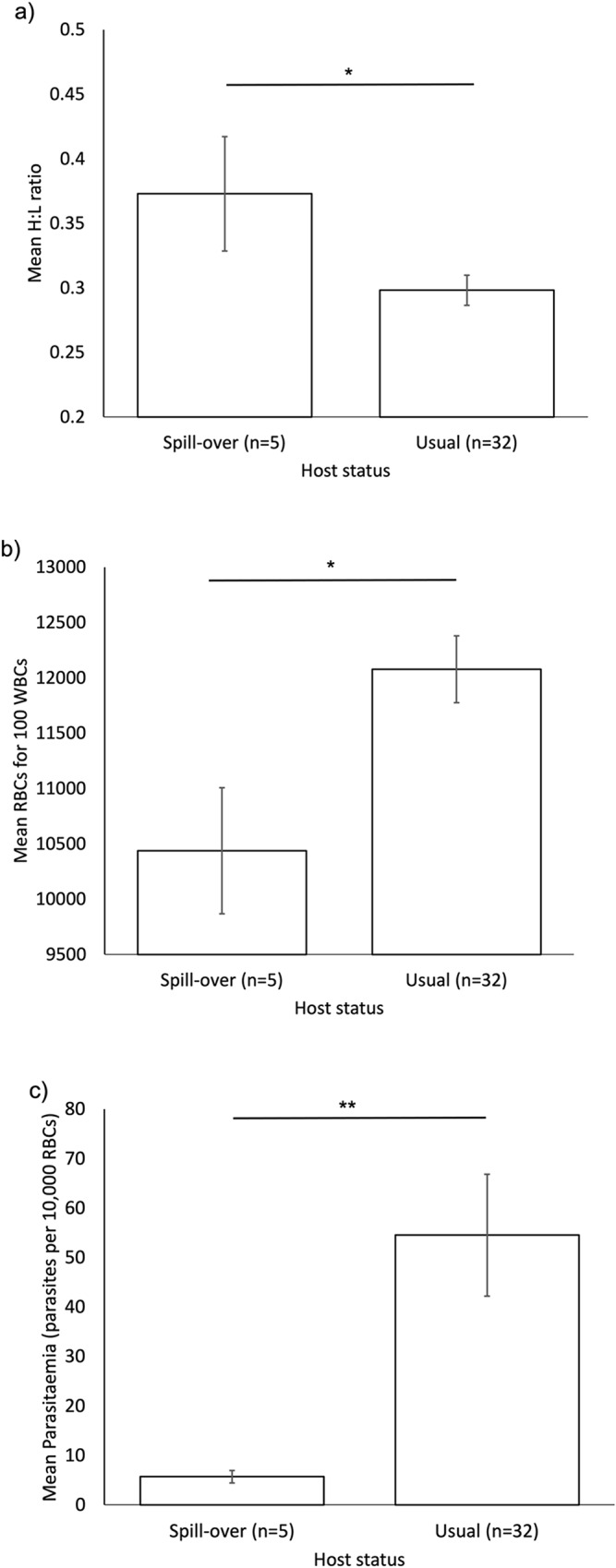
Differences in (a) H:L ratio, (b) RBC:WBC ratio and (c) parasitaemia between usual and spillover host species.

In RBC:WBC ratio and parasitaemia analyses, we identified 2 outlying points, which were removed from statistical analyses to ensure both normality of residuals, and that these points did not overestimate patterns of statistical significance. These points were retained within graphed data (Figures 1 and 2). Both points were from 2 dunnocks: one with an exceptionally high parasite load (477 parasites per 10 000 RBC; data range following removal of this point: 2–193 parasites per 10 000 RBC) and low RBC:WBC ratio (6512), and 1 uninfected with a high RBC:WBC ratio (18 337; range following removal of these 2 points: 8547–16 641).

Bars indicate mean values; error bars show ±1 SE. Significant differences are indicated above bars, with *P<0.05, **P<0.01, ***P<0.001.

Bars indicate mean values; error bars show ±1 SE. Significant differences are indicated above bars, with *P<0.05, **P<0.01, ***P<0.001.

We did not control for lineage identity or host species in any models due to autocorrelation with the terms of interest, because each specialist lineage was found mostly in a single host species (Supplementary Table 1), and each parasite lineage was classified as either specialist or generalist. Data were collected from 2 slides for most birds, so Bird ID was included as a random effect in all models to control for pseudoreplication.

## Results

### Parasite status effects on immune parameters and parasitaemia

H:L ratios differed with parasite status (LMM, 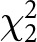 = 16.82, *P* < 0.001; Figure 1A), with no difference between birds infected by specialist parasites and uninfected birds (*t* = − 1.640, *P* = 0.10), but with birds infected by generalist parasites having a higher H:L ratio than both those infected by specialist parasites (*t* = −3.038, *P* = 0.003) and uninfected birds (*t* = −4.197, *P* < 0.001).

RBC:WBC ratios differed with parasite status (LMM, 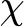
^2^_2_ = 11.10, *P* = 0.004; Figure 1B). There was no difference between birds infected by specialist parasites and uninfected birds (*t* = 1.14, *P* = 0.26), but birds infected by generalist parasites had lower RBC:WBC ratios than both those infected by specialist parasites (*t* = −2.64, *P* = 0.01) and uninfected birds (*t* = 3.41, *P* = 0.001).

Parasitaemia was higher in birds infected by specialist parasites than in those infected by generalist parasites (LMM, 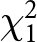 = 8.47, *P* = 0.004; Figure 1C).

### Usual vs spillover hosts

Spillover hosts had a higher H:L ratio than usual hosts (LMM, 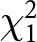 = 5.79, *P* = 0.02; Figure 2A) and a lower RBC:WBC ratio (LMM 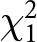 = 6.62, *P* = 0.01; Figure 2B). Parasitaemia in usual hosts was nearly 10 times higher than in spillover hosts (LMM 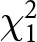 = 9.49, *P* = 0.002; Figure 2C), but gametocytes were observed in slides from all spillover hosts, confirming the presence of transmissible infection. Summary statistics for each parasite lineage are presented in Supplementary Table S2.

## Discussion

We found that both generalist parasites and specialist parasites in spillover hosts were associated with greater immune costs to their hosts. However, contrary to our predictions, parasitaemia was lower in both these 2 groups. This supports the idea that specialist parasites and usual hosts have adapted to one another resulting in reduced virulence and increased parasite reproduction rate, both of which act together to increase parasite transmission and reduce host costs (Leggett et al., 2013). However, both specialist parasites in spillover hosts, and generalist parasites seem to have increased virulence (as assessed through 2 measures of host immune response) and reduced parasite reproduction rate (as measured by parasitaemia). Elsewhere, it is generally accepted that a higher parasite reproductive success is associated with both a higher parasite virulence and increased transmission (Acevedo et al., 2019). However, our results appear to contradict this finding, with generalist *Haemoproteus* lineages having a larger effect on immune metrics (suggesting increased virulence) than specialist lineages, despite a much lower parasitaemia, in contrast to another study of haemosporidians that found higher parasitaemia in generalists (Huang et al., 2018).

We use parasitaemia here as a proxy for reproductive rate because only gametocytes (the reproductive stage of *Haemoproteus* parasites) are found within circulating erythrocytes (Pérez-Tris and Bensch, 2005; Valkiūnas, 2005). Gametocytes were identified in all slides examined, confirming host competence, so it is possible that the lower parasitaemia seen in generalist-infected hosts is still sufficient for transmission. Literature on the lower limits of transmissibility for *Haemoproteus* is sparse, but 0.5–1% is considered to be the optimal parasitaemia for natural transmission, based on experiments aiming to optimize vector survival and allow sufficient parasite sporogony within the vector stages for examination (Valkiūnas, 2005; Chagas et al., 2019). Indeed, it may be that high parasitaemias could be maladaptive for the parasite: these are associated with rapid mortality in biting midge (*Culicoides*) vectors (Valkiūnas and Iezhova, 2004; Bukauskaitė et al., 2016), although mortality reports from birds tend to be from dead-end infections of hosts where the parasite is unable to produce gametocytes and parasites are only found within organs (Donovan et al., 2008). However, the levels of parasitaemia we observed in both generalist- and specialist-infected birds are well below the level at which mortality is seen in vectors, at 0.1% and 0.5%, respectively (Valkiūnas, 2005). While the 0.1% seen in generalist-infected birds may fall below the range for optimal transmission, only 1 mature *Plasmodium* gametocyte is required to continue the life cycle within the vector (Bousema and Drakeley, 2011), and this is likely also the case for *Haemoproteus*. Therefore, it seems likely that transmission of both generalist and specialist lineages is possible in all hosts screened here.

We assess virulence here as immune response to infection, rather than mortality, using 2 different immune metrics. H:L ratio is commonly used as an indicator of stress in birds, with increased heterophil numbers (H:L ratio) reliably indicative of increased circulating glucocorticoid levels (Davis et al., 2008) and immune response (Krams et al., 2012). Generalist-infected birds and specialist-infected spillover hosts both display significantly elevated H:L ratios compared to other groups, strongly suggesting that these groups experience elevated stress in response to infection. This is similar to findings from other systems, such as House Finches *Carpodacus mexicanus* infected by *Mycoplasma gallisepticum*, where infected birds also exhibited elevated H:L ratios (Davis et al., 2004). The RBC:WBC ratio, or standardized WBC count, provides an indication of leukopenia (a decrease in WBCs) or leucocytosis (an increase in WBCs). An increase in WBCs compared to RBCs may also be indicative of anaemia (a reduction in RBCs) and needs to be interpreted carefully. Haemoparasites intrinsically cause anaemia through destruction of infected erythrocytes (Valkiūnas, 2005; Palinauskas et al., 2008), so if the pattern we see is driven by anaemia, then we should see an association between increased parasitaemia and a greater reduction in the RBC:WBC ratio. However, we actually see the opposite: specialist-infected birds have higher parasitaemia but a smaller reduction in RBCs, suggesting that the pattern we see may not be driven by a reduced RBC count, but in fact by an elevated WBC count; i.e. an increased immune response. This supports the suggestion of Garcia-Longoria et al. (2019) whereby the relationships of parasites with host species may be immune-modulated.

The higher parasitaemia seen in specialist lineages in their usual hosts compared to both generalists, and specialist lineages in spillover hosts may suggest an evolved tolerance to specialist lineages in their usual hosts. This concurs with findings from Galen et al. (2022) who found that specialist parasites were more likely than generalist parasites to be associated with host mortality, but more so when infecting spillover hosts at greater phylogenetic distance from their usual hosts (Galen et al., 2022). The evolution of tolerance to avian haemoparasites has been observed in previously susceptible species (Atkinson et al., 2013) and results in a reduction in negative fitness effects despite a given parasite load (Arriero et al., 2018). In support of this, in both spillover hosts for specialist lineages and generalist-infected hosts, we see elevated effects on immune metrics of a much lower parasite load, suggesting a higher cost to these hosts. Tolerance, as opposed to resistance, of parasite infection in animals is a relatively recent concept (Råberg et al., 2009), and more research into the relative roles of tolerance and resistance to different blood-borne parasites would be valuable (Rivero and Gandon, 2018). We caution that our sample size of spillover hosts is, by definition, small and encourage further work to corroborate this finding.

## Conclusions

Here, we use data from an avian haemoparasite community to test for differential effects of specialist and a generalist parasite lineage on multiple host species. Our data provide support for the suggestion of the evolution of tolerance in specialist host–parasite interactions, with increased transmission efficiency for the parasite (higher parasitaemia) and reduced impacts on the host (no difference in immune metrics compared to control birds). Conversely, in birds infected by a generalist lineage and spillover hosts for specialist lineages, we find greater immune response despite lower parasitaemia.

## Supporting information

Armour et al. supplementary material Armour et al. supplementary material

## Supplementary material

The supplementary material for this article can be found at https://doi.org/10.1017/S0031182025000393.
