# Supplementary material for: Do specialist haemoparasites induce tolerance in their hosts?
Source: Parasitology. 2025 Mar 26;152(4):374–80. doi: 10.1017/S0031182025000393 (PMC12186093; doi:10.1017/S0031182025000393)
Supplement: Armour et al. supplementary material [file S0031182025000393sup001.docx]

**Supplementary Material**

**Table S1.** Host-parasite associations for the lineages included in this study, along with Shannon index values for host diversity, SPD_i_ and PD_i_. Column headings are parasite lineages, row names are hosts; numbers are the numbers of times each lineage is found within each host.

| Species | | CARCHL01 | DUNNO01 | EMCIR01 | SYAT01 | SYAT02 | SYAT03 | TUPHI01 | TURDUS2 |
| --- | --- | --- | --- | --- | --- | --- | --- | --- | --- |
| Blackbird | *Turdus merula* | 1 | 2 |  |  |  |  |  | 19 |
| Blackcap | *Sylvia atricapilla* |  |  |  | 12 | 13 | 6 |  |  |
| Blue tit | *Cyanistes caeruleus* | 4 |  | 1 |  | 1 |  | 1 | 1 |
| Bullfinch | *Pyrrhula pyrrhula* |  |  | 1 |  |  |  |  |  |
| Chaffinch | *Fringilla coelebs* | 1 |  |  |  |  |  |  |  |
| Dunnock | *Prunella modularis* | 2 | 49 |  |  |  |  |  |  |
| Goldfinch | *Carduelis carduelis* | 3 |  |  |  |  |  |  |  |
| Great tit | *Parus major* |  |  |  |  |  |  |  |  |
| House sparrow | *Passer domesticus* | 1 |  |  |  |  |  |  |  |
| Linnet | *Linaria cannabina* | 4 |  |  |  |  |  |  |  |
| Reed bunting | *Emberiza schoeniclus* | 1 |  |  |  |  |  |  |  |
| Reed warbler | *Acrocephalus scirpaceus* |  |  | 1 |  |  |  |  |  |
| Robin | *Erithacus rubecula* | 2 |  | 1 |  |  |  |  |  |
| Sedge warbler | *Acrocephalus schoenobaenus* |  |  |  |  | 1 |  |  |  |
| Song thrush | *Turdus philomelos* |  |  |  |  |  |  | 11 |  |
| Whitethroat | *Sylvia communis* | 1 |  |  |  |  |  |  |  |
| Willow warbler | *Phylloscopus trochilus* |  | 1 |  |  |  |  |  |  |
| Wren | *Troglodytes troglodytes* | 2 |  |  |  |  |  |  |  |
| Yellowhammer | *Emberiza citrinella* | 1 |  | 19 |  |  |  |  |  |
| *Total* |  | 23 | 52 | 23 | 12 | 15 | 6 | 12 | 20 |
| Shannon index (H) |  | 2.329 | 0.257 | 0.703 | 0 | 0.485 | 0 | 0.287 | 0.199 |
| Taxonomic diversity (SPD_i_) | | 28.808 | 3.645 | 10.514 | 0 | 8.342 | 0 | 5.407 | 3.244 |
| Phylogenetic distinctness (PD_i_) | | 3.937 | 2.500 | 2.750 | 0 | 0.375 | 0 | 0.063 | 0.063 |
| Classification |  | Generalist | Specialist | Specialist | Specialist | Specialist | Specialist | Specialist | Specialist |

**Table S2.** Summary statistics for each parasite lineage included in this study. Statistics presented are mean ± 1 SE. H:L is the number of heterophils / (number of heterophils + number of lymphocytes), WBC:RBC ratio is: (estimated number of RBCs examined / number of WBCs examined) * 100, and parasitaemia is the number of parasites found divided by the number of RBCs examined, multiplied by 10,000, e.i. the number of parasites per 10,000 RBCs.

|  | CARCHL01 | DUNNO01 | EMCIR01 | SYAT01 | SYAT02 | SYAT03 | TUPHI01 | TURDUS2 |
| --- | --- | --- | --- | --- | --- | --- | --- | --- |
| H:L ratio | 0.36 ± 0.01 | 0.31 ± 0.02 | 0.33 ± 0.02 | 0.28 ± 0.02 | 0.26 ± 0.02 | 0.28 ± 0.03 | 0.29 ± 0.01 | 0.33 ± 0.02 |
| RBCs for 100 WBCs | 10810 ± 208 | 11304 ± 564 | 11895 ± 281 | 13876 ± 703 | 13065 ± 452 | 11150 ± 537 | 12038 ± 409 | 11552 ± 289 |
| Parasitaemia | 10.68 ± 1.17 | 81.49 ± 25.12 | 24.76 ± 4.01 | 32.14 ± 3.56 | 78.89 ± 15.15 | 114.91 ± 40.10 | 12.81 ± 1.57 | 23.82 ± 3.22 |
